# Late‐onset hemophagocytic lymphohistiocytosis with neurological presentation

**DOI:** 10.1002/ccr3.1135

**Published:** 2017-09-12

**Authors:** Sarah Benezech, Thierry Walzer, Emily Charrier, Damien Heidelberg, Geneviève De Saint‐Basile, Yves Bertrand, Alexandre Belot

**Affiliations:** ^1^ Department of Pediatrics Hospices Civils de Lyon Lyon France; ^2^ Institut National de la Santé et de la Recherche Médicale U1111 Université de Lyon 1 Lyon France; ^3^ Department of Radiology Hospices Civils de Lyon Lyon France; ^4^ Institut National de la Santé et de la Recherche Médicale U768 CHU Paris ‐ Hôpital Necker‐Enfants Malades Paris France; ^5^ Hospices Civils de Lyon Institut d'Hématologie et Oncologie Pédiatrique Lyon France; ^6^ Department of Rheumatology Hospices Civils de Lyon Lyon France

**Keywords:** Hemophagocytic lymphohistiocytosis, late‐onset, neurology, perforin

## Abstract

Missense mutations in genes involved in familial hemophagocytic lymphohistiocytosis can delay the onset of this life‐threatening disease. In children and adults, early recognition of aspecific features as neurological symptoms is crucial as urgent treatment is required.

## Introduction

Familial hemophagocytic lymphohistiocytosis (FHL) is the primary form of hemophagocytic lymphohistiocytosis (HLH). It is a rare and invariably fatal – without treatment – condition that takes onset in the early infancy. FHL results from a deficiency in cytotoxicity of CD8+ T lymphocytes (CTL) and Natural killer (NK) cells, causing an uncontrolled activation of T lymphocytes and macrophages and an inflammatory storm.

*PRF1* was the first gene associated with FHL 1. It is located on chromosome 10q21 and encodes perforin, a pore‐forming protein involved in cell death, expressed by CD8+ CTL and NK cells. *PRF1* mutations are associated with a defect in CD8+ CTL and NK Cells cytotoxicity, causing FHL type 2. Impairment of the cytotoxic function results in a defective clearance of antigen‐presenting cells and abnormally sustained antigen presentation. This leads to uncontrolled activation of T lymphocytes and subsequently macrophages and to secretion of high levels of proinflammatory cytokines.

According to the HLH‐2004 revised guidelines, the diagnosis of HLH can be established either if a molecular diagnosis is performed or if the patient fulfills five of the eight following diagnostic criteria: fever, splenomegaly, cytopenias, hypertriglyceridemia and/or hypofibrinogenemia, hemophagocytosis, low or absent NK cell activity, high ferritin level, and increased soluble IL‐2 receptor 2. Other clinical findings include liver dysfunction, lymphadenopathy, and neurological symptoms.

Familial hemophagocytic lymphohistiocytosis typically occurs during infancy, with an onset within the first year of life for about 70–80% of cases. Before the genetic and molecular diagnosis era, HLH that occurred in older children was usually thought as “secondary” HLH. During the last decade, a growing number of late‐onset authentic FHL cases have been reported, and it is now admitted that first manifestations of FHL can occur in elderly childhood and even adulthood.

We report here the case of a late‐onset FHL in a 7‐year‐old boy, with neurological presentation, associated with two new compound heterozygous mutations in *PRF1*.

## Case Report

**Figure 1:**
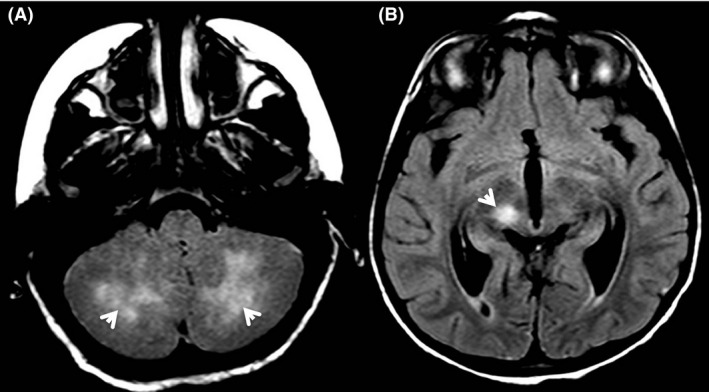
(A) Cerebellar lesions with moderate cerebral edema. (B) High‐intensity signal of right cerebral peduncle, substantia nigra, and red nucleus.

**Figure 2:**
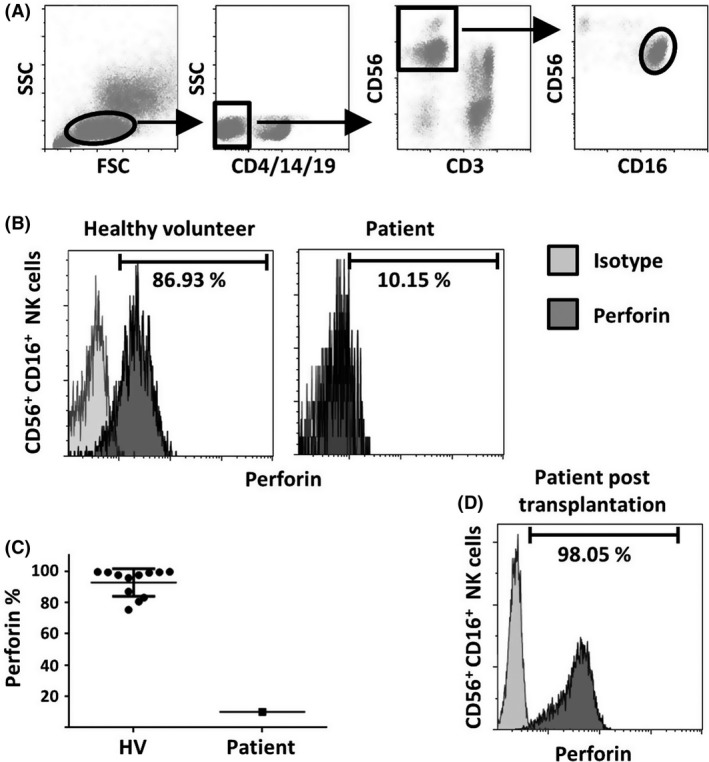
Deficient expression of perforin in patient. (A) Representative gating strategy for the identification of CD56+ CD16+ NK cells in which perforin is expressed. After exclusion of doublets and CD4+, CD14+ and CD19+ cells, NK cells are defined as CD3‐ CD56+ lymphocytes. Then, NK dim is identified by the expression of CD16 marker. (B) Representative histogram of perforin expression in CD56 dim NK cells from healthy volunteer (left) and patient (right). Percentage of CD56 dim NK cells expressing perforin is determined in comparison with an isotype control. (C) Dot plot graph comparison of the expression of perforin from CD56 dim NK cells in twelve healthy volunteer and in the patient. (D) Histogram of perforin expression in CD56 dim NK cells from patient after bone marrow transplantation. Percentage of CD56 dim NK cells expressing perforin is determined in comparison with an isotype control.

A 7‐year‐old boy was addressed to a hematologic tertiary hospital in a context of pancytopenia, asthenia, and persistent fever, few months after he presented an explosive varicella. He had no particular history. Physical exam found a hepatosplenomegaly. Except for the mild pancytopenia (neutrophils: 0.9 × 10^9^/L, hemoglobin: 95 g/L, platelets: 125 × 10^9^/L) and slightly elevated ferritinemia (102 *μ*g/L, N < 80), blood tests were virtually normal. Bone marrow aspiration revealed signs of hemophagocytosis without blastic cells. The first conclusion was a postviral “secondary” hemophagocytic lymphohistiocytosis episode, and no specific treatment was decided. Fever and asthenia persisted and a few weeks later he presented a diplopia due to the abducent nerve palsy, associated with a meningism and a cerebellar ataxia. Hepatosplenomegaly and polyadenopathy were present. Blood EBV, CMV, and HHV6 PCR were negative; there was a moderate Parvovirus B19 replication (600 copies/mL). EBV serology revealed a past infection with positivity for EBNA antigens. Pancytopenia persisted, fibrinogen was decreased, and ferritin was then increased up to 1800 *μ*g/mL. The patient had also circulating activated CD8 T cells (CD8 DR positive: 38%, *N* < 16). Cerebrospinal fluid analysis showed a white cell count <2, but after cytocentrifugation a lymphoid reaction with normal lymphocytes, some of them activated. A brain magnetic resonance imaging revealed multiple lesions in the cerebellar lobes and the substantia nigra (Fig. 1). Perforin expression in NK cells was decreased five‐ to tenfold compared to healthy individuals, as measured by flow cytometry (Fig. 2A–C). Genetic exams showed that the patient harbored two missense mutations in the *PRF1* gene: c.976T>C (p.S326P) and c.1130G>A (p.C377Y). His father and mother were respectively heterozygote for S326P and C377Y alleles. These mutations were not registered in the ExAC database 3 that screen more than 60,000 normal human exome sequencing. The pathogenicity of these mutations was assessed according to PolyPhen‐2 and SIFT software with respectively 0.998 and 0.999 (PolyPhen‐2) and 0.17, and 0 (SIFT) scores. Patient’s NK cell degranulation was normal when PBMC was stimulated with K562 target cells, excluding the possibility that mutations in genes involved in granule exocytosis also contributed to the disease (data not shown). Patient was treated according to the current national protocol 4. A corticotherapy (2 mg/kg/day) was started that rapidly improved the systemic and neurological symptoms. Then a treatment with cyclosporin was started. While the corticosteroid was being decreased to 0.5 mg/kg/day the patient relapsed, presenting a hemiparesis and an intracranial hypertension. He received an antithymocyte globulin (ATG) treatment that was quickly switched for alemtuzumab because of side effects and deterioration of the neurological status. Clinical and radiological features improved, and he underwent bone marrow transplantation (BMT) from a 9/10 HLA‐matched unrelated donor. Perforin expression was normalized after BMT (Fig. 2D). The child was alive and well at 12‐month‐post BMT with full complete chimerism.

## Discussion

**Figure 3:**
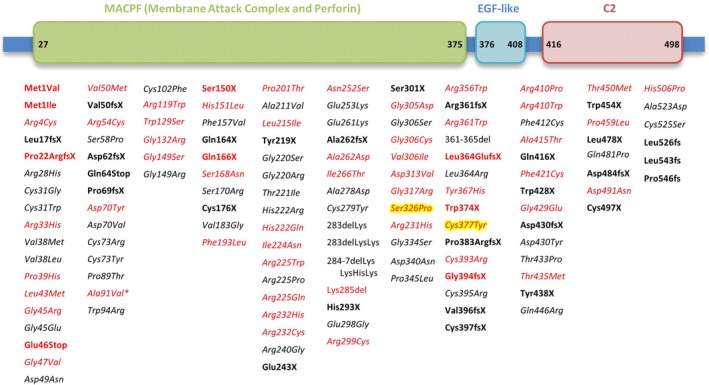
Mutations in *PRF1* reported in literature. Bold: nonsense mutation; normal: deletion; italic: missense mutation; *: variant with controversial pathogenicity; highlighted: mutation reported here; red: late‐onset associated mutation.

To date, there are more than 100 *PRF1* mutations reported in literature (Fig. 3, references in Appendix 1). The two mutations presented by our patient have not been reported previously. The variants c.976T>C (p.S326P) and c1130G>A (p.C377Y) are therefore new missense mutations prone to cause familial hemophagocytic lymphohistiocytosis. Unlike nonsense *PRF1* mutations, missense mutations in homozygous or compound heterozygous status may be associated with a residual function of the perforin protein, with a delayed age of onset 5. When a nonsense mutation is described in late‐onset FHL, it is found to be either single allele mutation or associated with a missense mutation. Homozygosity or compound heterozygosity with nonsense mutations consistently leads to an early‐onset disease. Regarding late‐onset FHL, the hypothesis of a second hit mechanism is more and more admitted to explain the disparity between ages of onset. Viruses could play this key role, as EBV infection is frequently reported to go along with the onset of FHL. But viral infection is not a constant finding and several authors assume that this second hit could also be a molecular event involving the cytotoxicity pathway.

CNS (central nervous system) involvement is a common finding in FHL course 6, 7, although not a risk factor for poor outcome, as recently reported 8. The neurological semiology is nonspecific and polymorphous, due to cytokines and inflammatory cells infiltration: the most frequent manifestation is an aseptic meningitis, but CNS disease can also be suspected in the case of cranial nerve palsies, ataxia, seizures, or coma. The case we report is another proof that the onset of FHL is frequently associated with CNS involvement. Through literature, we noticed many patients were misdiagnosed because of predominant neurological symptoms that may precede systemic manifestations. For instance, among late‐onset FHL cases, several patients were first diagnosed and treated for demyelinating disorders. This frequent but unusual neurological presentation for a life‐threatening hematologic disease can delay the diagnosis and worsen the prognosis. Moreover, as delayed as could be the onset of FHL, the prognosis seems to be as poor as in the classical infant form, unless an hematopoietic stem cells transplantation is performed. Thus, faced with presumed “post‐viral” features, the early recognition of a potential genetic immune deficiency is crucial to improve the prognosis of these patients.

## Authorship

SB: Drafted and revised manuscript for intellectual content. TW: Analyzed and interpreted data. EC: Analyzed and interpreted data. DH: Analyzed and interpreted data. GDSB: Analyzed and interpreted data. YB: Involved in critical revision of manuscript for intellectual content. AB: Involved in draft supervision and critical revision for intellectual content.

## Conflicts of Interest

None declared.
